# Insights into aging from progeroid syndrome epigenetics

**DOI:** 10.18632/aging.204977

**Published:** 2023-08-05

**Authors:** Yosra Bejaoui, Junko Oshima, Nady El Hajj

**Affiliations:** 1 College of Health and Life Sciences, Hamad Bin Khalifa University, Qatar Foundation, Qatar; 2 Department of Laboratory Medicine and Pathology, University of Washington, Seattle, WA 98105, USA; 3 Department of Clinical Cell Biology and Medicine, Graduate School of Medicine, Chiba University, Chiba, Japan; 4 College of Science and Engineering, Hamad Bin Khalifa University, Qatar Foundation, Doha, Qatar

**Keywords:** premature aging, epigenetics, progeroid syndromes, epigenetic clocks, DNA methylation

Human progeroid syndromes are characterized by an accelerated aging phenotype mimicking normal aging. The term *progeria *originated from the Greek word “*pro*” and the word “*geras*”, which mean “before old age”. In ancient Greek mythology, “Geras” was the name of the god of old age. Progeroid syndromes can be classified into segmental progeroid syndromes, in which the senescent phenotype affects more than one organ or tissue, or unimodal progeroid syndromes, in which the accelerated aging phenotype only affects one tissue. Despite intensive research efforts leading to the discovery of single-gene mutations in the majority of progeroid syndromes, the relationship between genotypes and the accelerated aging phenotypes remains poorly understood. Hutchinson–Gilford Progeria Syndrome (HGPS) is one of the most severe forms of premature aging that belongs to a group of disorders known as laminopathies, which are caused by mutations in the *LMNA* gene [1]. Named after Dr. James Hutchinson and Dr. Hastings Gilford who first described the disease in 1886 and 1897, respectively, HGPS is used as a model to study aging, contributing to our understanding of how defects in the nuclear architecture can lead to genome instability and premature aging [2].

In this editorial, we discuss “DNA methylation signatures in Blood DNA of Hutchinson–Gilford Progeria syndrome” [3]. Our study revealed no differences in DNA methylation age acceleration as measured by epigenetic clocks in HGPS patients with classical (1824 C>T) and non-classical mutations in *LMNA. *A significant hypomethylation of solo-WCGW CpG sites in partially methylated domains (PMDs) was observed in our study of HGPS. Solo-WCGW CpG sites are CpG sites flanked by an Adenine or Thymine (W) and their hypomethylation signature in PMDs is considered a barometer of cell proliferation and aging-related methylation loss [4]. Furthermore, comparing differentially methylated sites in progeroid laminopathies, Werner syndrome, and Down syndrome (also considered a segmental progeroid syndrome), we found a common epigenetically dysregulated region that regulates a long non-coding RNA anti-sense to the Catenin Beta Interacting Protein 1 gene (*CTNNBIP1*), which is a negative regulator of Wnt signaling. As dysregulation of WNT signaling has been implicated in aging, understanding how this lncRNA regulates *CTNNBIP1* and the Wnt signaling pathway may provide insights into the molecular mechanisms underlying premature aging.

Interestingly, HGPS patients did not show any significant increase in epigenetic age acceleration considering they display a drastic aging phenotype. One explanation could be related to the rapid age acceleration and short lifespan of HGPS patients (~13 years), which does not allow sufficient time for the epigenome to reflect the premature aging phenotype. This is in contrast to Werner syndrome, known as adult progeria, since the symptoms of the disease manifest when patients are in their 20s and 30s [5]. A recent report suggests that epigenetic aging is not linked to the aging hallmarks associated with cellular senescence, genomic instability and telomere shortening. However, there may be a connection between epigenetic aging and hallmarks related to stem cell composition, mitochondrial activity, and nutrient sensing [6]. This could explain the observed normal epigenetic aging in HGPS patients, which also aligns with the work performed by our labs on two disorders with clinical features of accelerated aging: Berardinelli–Seip Congenital Lipodystrophy type 2 (CGL2) and Cerebroretinal microangiopathy with calcifications and cysts (also known as Coats plus syndrome) [7]. CGL2 is caused by mutations in the Berardinelli-Seip Congenital Lipodystrophy 2 (*BSCL2*) gene, which leads to the most severe form of congenital lipodystrophy. Despite being non-obese, CGL2 patients suffer from severe metabolic complications including insulin resistance and diabetes. In those patients, we could observe strong epigenetic age acceleration as measured by different epigenetic clocks, which could be related to a premature aging phenotype linked to the nutrient sensing hallmark [8]. On the other hand, epigenetic age measurement in Coats plus syndrome patients did not reveal DNA methylation age acceleration. This similarly aligns with the observation that epigenetic clocks do not measure changes related to telomere attrition. Coats plus syndrome is caused by premature telomere shortening due to pathogenic variants in the conserved telomere maintenance component 1 (*CTC1*) gene, which is an essential component of the CST complex that is involved in telomere maintenance and protecting telomeres from degradation.

The force of selection weakens as an organism ages; therefore, natural selection may not have the opportunity to select for genes that prolong longevity and health at an older age. Although those genetic variants are not common, “experiments of nature,” in which genetic mutations cause extreme aging phenotypes, can help us discover genes and pathways linked to successful aging and longevity. This avenue involves focusing on progeroid syndromes, whereas the other avenue involves focusing on “unimodal antigeroid syndromes,” which would entail studying and identifying genetic variants that protect against aging-related disorders.
